# Polyhydroxyalkanoate involvement in stress-survival of two psychrophilic bacterial strains from the High Arctic

**DOI:** 10.1007/s00253-024-13092-8

**Published:** 2024-03-23

**Authors:** Jakub Grzesiak, Małgorzata Marta Rogala, Jan Gawor, Xenie Kouřilová, Stanislav Obruča

**Affiliations:** 1 Institute of Biochemistry and Biophysics, Polish Academy of Sciences, Pawińskiego 5A, 02-106 Warsaw, Poland; 2 Department of Food Chemistry and Biotechnology, Faculty of Chemistry, Brno University of Technology, Purkynova 118, 612 00 Brno, Czech Republic

**Keywords:** Polyhydroxyalkanoates, Survival, Environmental stressors, Arctic bacteria

## Abstract

**Abstract:**

An ever-growing body of literature evidences the protective role of polyhydroxyalkanoates (PHAs) against a plethora of mostly physical stressors in prokaryotic cells. To date, most of the research done involved bacterial strains isolated from habitats not considered to be life-challenging or extremely impacted by abiotic environmental factors. Polar region microorganisms experience a multitude of damaging factors in combinations rarely seen in other of Earth’s environments. Therefore, the main objective of this investigation was to examine the role of PHAs in the adaptation of psychrophilic, Arctic-derived bacteria to stress conditions. Arctic PHA producers: *Acidovorax* sp. A1169 and *Collimonas* sp. A2191, were chosen and their genes involved in PHB metabolism were deactivated making them unable to accumulate PHAs (Δ*phaC*) or to utilize them (Δ*i-phaZ*) as a carbon source. Varying stressors were applied to the wild-type and the prepared mutant strains and their survival rates were assessed based on CFU count. Wild-type strains with a functional PHA metabolism were best suited to survive the freeze–thaw cycle — a common feature of polar region habitats. However, the majority of stresses were best survived by the Δ*phaC* mutants, suggesting that the biochemical imbalance caused by the lack of PHAs induced a permanent cell-wide stress response thus causing them to better withstand the stressor application. Δ*i-phaZ* mutants were superior in surviving UV irradiation, hinting that PHA granule presence in bacterial cells is beneficial despite it being biologically inaccessible. Obtained data suggests that the ability to metabolize PHA although important for survival, probably is not the most crucial mechanism in the stress-resistance strategies arsenal of cold-loving bacteria.

**Key points:**

• *PHA metabolism helps psychrophiles survive freezing*

• *PHA-lacking psychrophile mutants cope better with oxidative and heat stresses*

• *PHA granule presence enhances the UV resistance of psychrophiles*

**Supplementary Information:**

The online version contains supplementary material available at 10.1007/s00253-024-13092-8.

## Introduction

Microorganisms inhabit most of the available environments on Earth. They owe their successful colonization skills to the ability to withstand and adapt to environmental stressors (Gupta et al. 2017). The ability to store intracellular carbon-containing macromolecular polymers known as polyhydroxyalkanoates (PHAs) has in recent years been proven to be involved in coping with several damaging factors in many bacterial species (Obruca et al. 2020). Their main role in energy and carbon storage, helping to survive periodic carbon deficits, may therefore be challenged in light of the latest discoveries (Müller-Santos et al. 2021).

PHAs are most commonly accumulated by prokaryotic organisms (Bacteria and Archaea) in conditions described as “unbalanced,” when the growth environment is rich in labile carbon sources while simultaneously being relatively poor in available nitrogen (López et al. 2015). PHAs are large molecules (50 to 1000 kDa). They are composed of polyester of hydroxyalkanoic acids of varying length (Reddy et al. 2003). Insoluble and therefore osmotically inactive, they form in the shape of rounded granules of different sizes and quantities per cell (Shen et al. 2019). They accumulate via the combination of basic metabolic pathways (Krebs cycle, β-oxidation, etc.) and specialized reactions where the polymerization process is the most crucial one (Khatami et al. 2021). It is accomplished by the actions of PHA synthases (coded by the *phaC* gene) of which four classes were described in detail to date. Based on the chemical composition of produced PHAs two groups of synthases can be distinguished: classes I, III, and IV catalyze the polymerization of PHAs with short-length (scl-PHA, C3–C5 per monomer) side chains while class II synthases catalyze PHAs with medium-length (mcl-PHA, C6-C14) side chains (Neoh et al. 2022; Koller 2018). Usually, only one class of synthase exists within the genome of a particular bacterial strain; however, some exceptions were discovered (Tan et al. 2020). A bacterial cell can access its PHA-stored carbon source by employing PHA-degrading enzymes of which the intracellular PHA depolymerase, coded by the *i-phaZ* gene, is first to start and is indispensable for the process (Knoll et al. 2009). Usually, the process of polymerization and depolymerization occurs simultaneously in the bacterial cell, causing a pool of soluble, free hydroxyacids to exist beside the PHA inclusions. This balance can be shifted toward one of those processes in response to changing environmental conditions (Ren et al. 2009).

An ever-growing body of literature evidences the protective role of PHAs against a plethora of mostly physical stressors including low temperature and freezing, heat shock, osmotic pressure, oxidation, UV irradiation, and exposure to high heavy metal concentrations (Obruca et al. 2020; Müller-Santos et al. 2021). As for some factors like UV exposure, the mechanism of PHA-based protection is fairly simple and understood; for others, it remains elusive and involved in complex multicomponent interactions (Slaninova et al. 2018; Obruca et al. 2018). Primary analysis on PHA involvement in stress survival of cells is based on exposure of PHA-containing and PHA-absent prokaryotic cells to physical and chemical-type stressors of varying intensities and comparing their ability to grow on microbiological media and/or to measure the damage caused to different cell components (Kim et al. 2013; Sedlacek et al. 2019b). To date, most of the research done involved mesophilic microorganisms, model strains isolated from habitats not considered to be life-challenging nor extremely impacted by abiotic environmental factors. Only a handful of studies were based on some type of extremophilic prokaryotic strain (Obruca et al. 2020).

Therefore, the main objective of this investigation was to examine the role of PHAs in the adaptation of psychrophilic, Arctic-derived bacteria to various stress conditions. Microorganisms inhabiting high-latitude/high-altitude sites (polar and alpine regions) experience a multitude of life-challenging factors in combinations rarely seen in other of Earth’s environments (Rhodes et al. 2013). This likely causes an evolutionary emergence and persistence of mechanisms enabling survival and growth in such harsh circumstances, presumably also the ability to store and utilize PHAs (Thomas et al. 2008). Our hypothesis states that PHA presence in cells of examined bacteria of Arctic origin contributes to their resistance to several damaging factors occurring in their native habitats such as freezing, UV radiation, and osmotic or oxidative pressure. This investigation combines molecular and classic microbiology aspects that gain insight into the mechanisms behind life’s ecological success, while also expanding the still scarce knowledge on polar region microbes and their unique adaptive traits in frequently changing and harsh conditions.

## Materials and methods

### Bacterial strains, plasmids, and culture conditions

The bacterial strains and plasmids used in this study are listed in Table 1. Arctic isolates *Acidovorax* sp. A1169 and *Collimonas* sp. A2191 were obtained from the Central Collection of Strains of the Institute of Biochemistry and Biophysics, Polish Academy of Sciences (http://kolekcja.ibb.waw.pl/). The strains were deposited at the publicly accessible Polish Collection of Microorganisms (PCM), culture no. 3255 (A1169) and 3256 (A2191). They were used as wild-type, low-temperature PHA producers (Rogala et al. 2020). *Escherichia coli* strain DH10B was used for plasmid transformation and propagation, while S17-1 was used for mobilization of the suicide plasmid pAKE604 into Arctic strains. Wild-type strains of *Acidovorax* sp. A1169, *Collimonas* sp. A2191, and their gene knockout mutants were cultured in R3A medium (1 g/L tryptone, 1 g/L peptone, 1 g/L beef extract, 1 g/L yeast extract, 1 g/L K_2_HPO_4_, 0.5 g/L NaH_2_PO_4_, 0.5 g/L Na-pyruvate, 0.1 g/L MgSO_4_‧7H_2_O) at 15 °C (if not otherwise indicated). All *E*. *coli* strains were cultured in LB broth on a shaker at 200 rpm and 37 °C or on LB agar at 37 °C. Where required, kanamycin was added to a final concentration of 25 or 50 mg/L to ensure plasmid maintenance and selection.

**Table 1:** Bacterial strains and plasmids used in this study

Strain/plasmid	Genotype/phenotype	Source/reference
*Acidovorax* sp. A1169		
Wild-type	PHB producer	Arctic glacier/Rogala et al. (2020)
Δ*phaC*	*phaC* gene knockout mutant derived from A1169; PHB-	This study
Δ*i-phaZ*	*i-phaZ* gene knockout mutant derived from A1169	This study
*Collimonas* sp. A2191		
Wild-type	PHB producer	Arctic tundra/Rogala et al. (2020)
Δ*phaC*	*phaC* gene knockout mutant derived from A2191; PHB-	This study
Δ*i-phaZ*	*i-phaZ* gene knockout mutant derived from A2191	This study
*E. coli*		
DH10B	F– mcrA Δ(mrr-hsdRMS-mcrBC) φ80lacZΔM15 ΔlacX74 recA1 endA1 araD139 Δ(ara-leu)7697 galU galK λ– rpsL(StrR) nupG	Thermo-Fisher Scientific
S17-1	recA pro hsdR RP4-2-Tc::Mu-Km::Tn7,λ-pir; mobilizer strain	Lab stock
Plasmid pAKE604	ori_MB1_ ori_TRK2_ Ap^r^ Km^r^ lacZ sacB	El-Sayed et al. (2001)

### DNA isolation and sequencing

Genomic DNA was isolated by the CTAB method (Wilson 2001). Plasmid isolation was performed with the Plasmid Midi AX or the Plasmid Mini kits (A&A Biotechnology) while DNA purification was conducted with the Clean-up Concentrator kit (A&A Biotechnology) according to manufacturer’s instructions. The genomes of *Acidovorax* sp. A1169 and *Collimonas* sp. A2191 were sequenced using an Illumina MiSeq apparatus (Illumina Inc., USA). The Illumina paired-end sequencing library construction was performed with 1 μg of post-nebulized DNA extract and the KAPA Library Preparation Kit reagents (KAPA Biosystems, USA), according to the manufacturer’s instructions. The library was pooled and sequenced on a MiSeq platform using the 600-cycle MiSeq reagent Kit v.3 (Illumina, USA). Sequence quality metrics were assessed using FASTQC (Andrews 2010).

### Genome assembly, annotation, primer design, PCR amplification, and cloning

Raw sequencing reads were trimmed for quality and residual library adaptors were removed using fastp software (Chen et al. 2018; https://academic.oup.com/bioinformatics/article/34/17/i884/5093234). Cleaned Illumina reads were assembled into contigs using SPAdes software (https://github.com/ablab/spades). Draft genomes were annotated using the BV-BRC platform (https://www.bv-brc.org/). SnapGene software (www.snapgene.com) was used to design primers for the PCR (Online Resource 1). PCR amplifications were performed using Mix Plus, PCR Mix Plus HGC, and PCR Mix RAPID ready-to-use mixes for PCR (A&A Biotechnology). Appropriate flanking region pairs were cloned into pAKE604 (Km^r^) vectors using the Anza Restriction Enzyme Cloning System (Thermo Fisher) according to the manufacturer’s instructions and then transformed into *E. coli* DH10B chemically competent cells made using the Inoue method (Inoue et al. 1990). Transformants were checked by colony PCR using specific primers (Online Resource 1).

### Bacterial conjugation

Recombinant plasmids were introduced into *E. coli* S17-1. Biparental mating with psychrophilic PHA producers was done as follows: saturated cultures of the respective PHA producer and *E. coli* S17-1 were washed with PBS and combined in a 3:1 ratio. The resulting suspension was drop-plated onto Conjugation Agar containing 1 g/L tryptone, 1 g/L peptone, 1 g/L beef extract, 1 g/L yeast extract, 1 g/L K_2_HPO_4_, 0.5 g/L NaH_2_PO_4_, 0.5 g/L Na-pyruvate, 0.1 g/L MgSO_4_‧7H_2_O, 3 g/L HEPES, 3 g/L NORIT® activated charcoal, and 15 g/L agar; pH was adjusted to 7.2 with 0.1 M KOH and 0.1 M HCl using Hanna pH meter. Plates were incubated at 15 °C for 48 h, after which the growth was scraped, serially diluted and plated onto R3A plates with kanamycin (25 mg/L), and incubated at 10 °C until single colonies developed (low temperature was used as a selection factor for psychrophilic transconjugants). Colonies of *K*m-resistant psychrophiles were picked, inoculated into R3A broth supplemented with 2.5% sucrose, and incubated at 10 °C with shaking until bacterial growth was apparent. The resulting suspension was diluted and plated onto R3A plates with 2.5% sucrose and incubated at 15 °C until colony development. Subsequently, they were screened for the target sequence by PCR, using appropriate primers (Online Resource 1).

### PHA production

PHA production in *Acidovorax* sp. A1169 and *Collimonas* sp. A2191 was performed according to Kourilova et al. (2021) with modifications. An active inoculum of wild-type and deletion mutants was prepared (R3A broth, 15 °C, 72 h) and added (10% v/v) to the PHA production medium. The PHA production medium consisted of the following: 9 g/L Na_2_HPO_4_·12H_2_O, 1.5 g/L KH_2_PO_4_, 1 g/L NH_4_Cl, 0.5 g/L yeast extract, 0.2 g/L MgSO_4_·7 H_2_O, 0.02 g/L CaCl_2_·2H_2_O, and 1 mL/L SL-11 trace element solution (5.2 g/L Na_2_-EDTA, 1.5 g/L FeCl_2_·4H_2_O, 190.0 mg/L CoCl_2_·6H_2_O, 100.0 mg/L MnCl_2_·4H_2_O, 70.0 mg/L ZnCl_2_, 36.0 mg/L Na_2_MoO_4_·H_2_O, 24.0 mg/L NiCl_2_·6H_2_O, 6.0 mg/L H_3_BO_3_, 2.0 mg/L CuCl_2_·2H_2_O). After autoclaving a 40% filter-sterilized fructose solution was added to the medium to a final concentration of 10 g/L. After 96 h of incubation at 15 °C and 125 rpm cells were harvested for further analysis.

### Application of stressors

To assess the wild-type strains and deletion mutants’ ability to access intracellular PHA as carbon and energy source the bacteria were subjected to PHA-accumulation conditions as described earlier. So prepared cells were washed with the PHA production medium without the primary carbon source and then introduced into the same medium at 10% v/v. Cell growth was monitored during the 96-h incubation period by colony-forming unit count on R3A agar at 15 °C.

The application of varying stressors was adjusted for each of the two bacterial species. Stressor intensity was considered sufficient when the CFU count of at least one of the strains of a particular species was decreased by ≥ 90%.

#### Thermal stress assay

Bacterial cultures after 96-h incubation at 15 °C in PHA production medium were diluted in PBS solution to a concentration of 10^7^ cells/mL in glass test tubes. The test tubes were placed in a water bath at 40 °C and incubated for 5 min (*Acidovorax* sp. A1169) or 15 min (*Collimonas* sp. A2191). Afterward, the tubes were placed in ice-chilled water for 10 min, serially diluted in PBS, plated on R3A agar, and incubated for 120 h at 15 °C to determine the number of colony-forming units.

#### Oxidative stress assays

For *Acidovorax* sp. A1169 sensitivity to H_2_O_2_ was measured by subjecting bacterial suspensions (10^7^ cells/mL of PBS) to a 15 mM H_2_O_2_ for 5 min, whereas for *Collimonas* sp. A2191 a concentration of 30 mM was applied for 20 min, immediately after bacterial suspensions were serially diluted and CFU numbers were assessed as described earlier.

#### Osmotic fluctuation assay

Osmotic stress was applied as described in Sedlacek et al. with modifications (2019b). For *Acidovorax* sp. A1169 the cells were harvested by centrifugation (3000 rpm for 5 min) and suspended in an equal volume of 10% NaCl solution for 30 min; afterward, the suspension was serially diluted in ddH_2_O and incubated for 30 min at 4 °C and plated onto R3A agar. For *Collimonas* sp. A2191 a 20% NaCl solution was used and a 1-h incubation period in both cases.

#### Acidic pH stress assay

Acid stress was applied to the bacterial strains by harvesting the cells by centrifugation and suspending them in citric acid buffer. *Acidovorax* sp. A1169 cells were suspended in pH 4.0 buffer for 5 min while *Collimonas* sp. A2191 cells were suspended in pH 3.0 buffer for 1 min. After the given time period, the cells were serially diluted in PBS buffer and CFU count was assessed as stated earlier.

#### UV irradiation stress assay

Three milliliters of bacterial suspension (10^7^ cells/mL of PBS) was poured onto a small Petri dish (50-mm diameter) and irradiated with UV (254 nm) using a UV cross-linker (UVP model CL-1000), with 30 kJ/m^2^ exclusively for *Acidovorax* sp. A1169 and 20 kJ/m^2^ exclusively for *Collimonas* sp. A2191. Bacterial suspensions were serially diluted before plating on R3A agar plates for manual CFU enumeration.

### Microscopy

Bacterial suspension (10 µL) was collected after the incubation period in a PHA production medium, placed on a microscope slide, air dried, and fixed by heating in a Bunsen burner flame. The so-fixed smear was stained with a SYBR-Nile Red solution in 50% methanol for 15 min. Afterward, the glass slide was rinsed with ddH_2_O and air-dried in darkness. A cover slide was fixed on top of the smear with a drop of CitiFluor AF1 anti-fade solution. Cells were observed under 1000 magnification with green (510–560 nm) and blue light (450–490 nm) excitation on a Nikon E-200 microscope with a 100-W Hg lamp and 100 × CFI 60 oil immersion objective. Images were captured with a digital DS-Fi3 high-definition color microscope camera equipped with a 5.9-megapixel CMOS image sensor and a filter block of wavelengths: EX 330–380, DM 400, BA 420.

### Biomass analysis

Bacterial suspension (10 mL) was collected after the incubation period on PHA production medium, centrifuged at 6000 × *g* for 5 min, washed with distilled water, and dried at 80 °C until constant mass was achieved. PHA composition and content of the dried biomass were determined by gas chromatography with a flame ionization detector (GC-FID) as described previously (Obruca et al. 2013). The PHA content in cells and the monomer composition of PHA were analyzed as methyl esters of particular 3-hydroxyacids by gas chromatography (GC) with flame ionization detector. Approximately 8 mg of dry cells was reacted in a small crimp neck vial (2 mL) with a solution containing 1 mL of chloroform and 0.8 mL of 15% sulfuric acid in methanol with benzoic acid (5 mg/mL) as an internal standard for 3 h at 94 °C in a dry bath heater. This method degrades the intracellular PHA by methanolysis to its constituent hydroxycarboxylic acid methyl esters. After the reaction, the entire content of the crimp neck vial was poured into screw-cap vial (4 mL) containing 0.5 mL of 0.05 M NaOH and the vial was shaken vigorously. After phase separation, 50 µL of the organic phase (bottom layer) was transferred to a small screw-cap vial (2 mL) containing 900 µL of isopropyl alcohol. The methyl esters were assayed by Trace GC Ultra, Thermo Fisher Scientific equipped with a Stabilwax (Restek) column (30 m × 0.32 mm × 0.5 µm) and a flame ionization detector. Standard calibration curves were prepared from commercially available polymers. The total PHA per cell dry weight was determined by summing the absolute amount of all of the hydroxyalkanoate monomer units detected.

### Data analysis

Illumina reads were deposited in the NCBI Sequence Read Archive (SRA) as BioProject PRJNA991094. All results were compiled using Excel 2016 (MS Office) for Windows. Data visualization and statistical analysis have been performed using the R software (R v.4.2.3) and the following packages: ggplot2 and ggpubr (R Core Team 2021). Phylogenetic trees were made using the Mega-X software using Blastn, Blastp, and DED databases (Altschul et al. 1990; Knoll et al. 2009).

## Results

### Taxonomic classification and genome characteristics of the psychrophilic strains

**Fig. 1:**
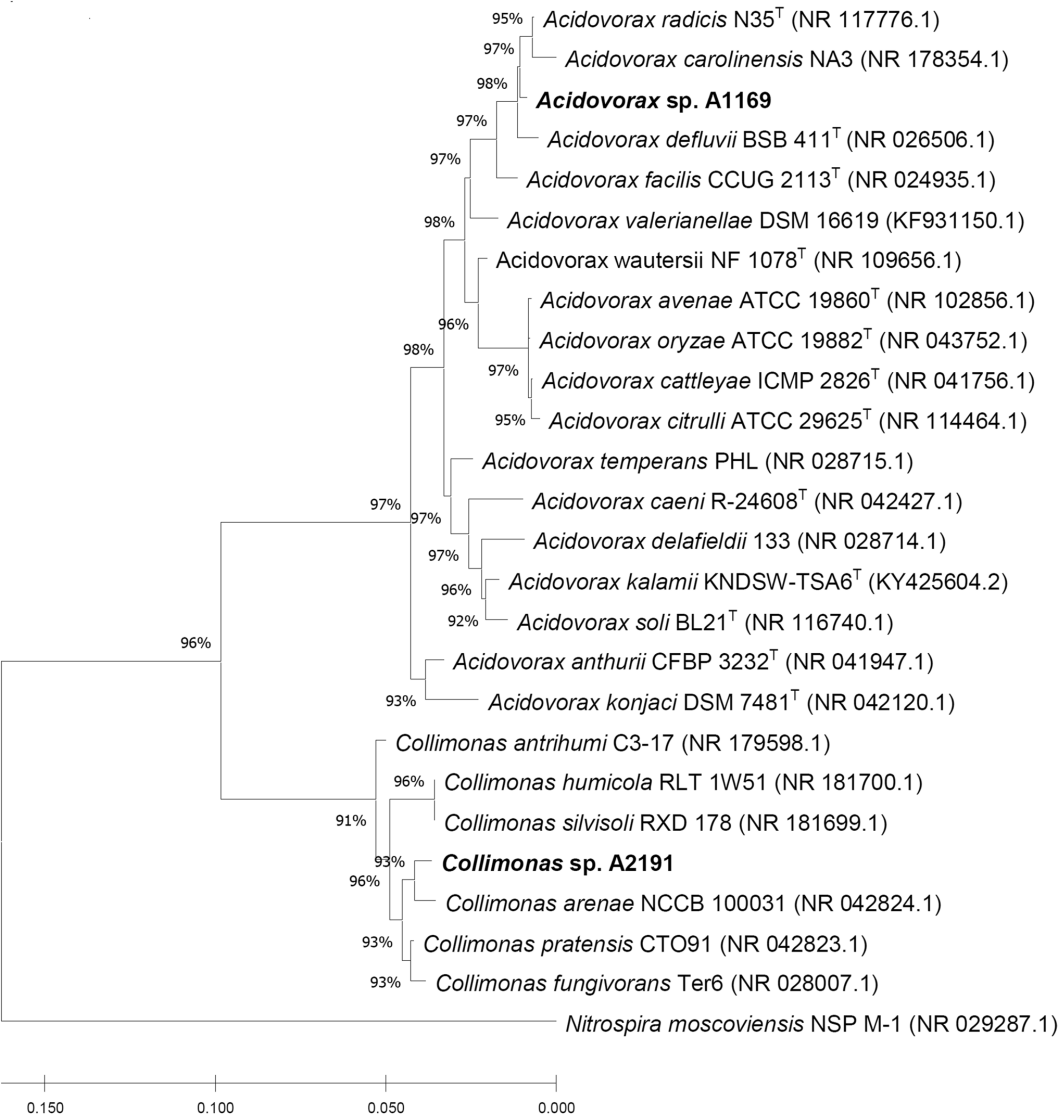
Neighbor-joining phylogenetic tree based on 16S rRNA gene sequences showing the position of strain A1169 and related species in the genus *Acidovorax* (family *Comamonadaceae*) and of strain A2191 and related species in the genus *Collimonas* (family *Oxalobacteraceae*). Numbers at nodes are bootstrap percentages based on the neighbor-joining algorithm. *Nitrospira moscoviensis* NSP M-1 was used as an outgroup. Sequences were retrieved from the NCBI database. Bar shows substitutions per nucleotide position

The genome sequencing-obtained 16S rRNA gene sequences of strain A1169 and A2191 displayed the greatest similarity with *Acidovorax radicis* (99.4% similarity) and *Collimonas pratensis* (98.9% similarity), respectively. However, the neighbor-joining algorithm placed strain A1169 closer to *Acidovorax carolinensis*, whereas A2191 closer to *Collimonas arenae*. The placement of those sequences on a phylogenetic tree comprising 16S rRNA gene sequences of validly published species indicates that they could be novel species within their respective genera (Fig. 1).

**Fig. 2:**
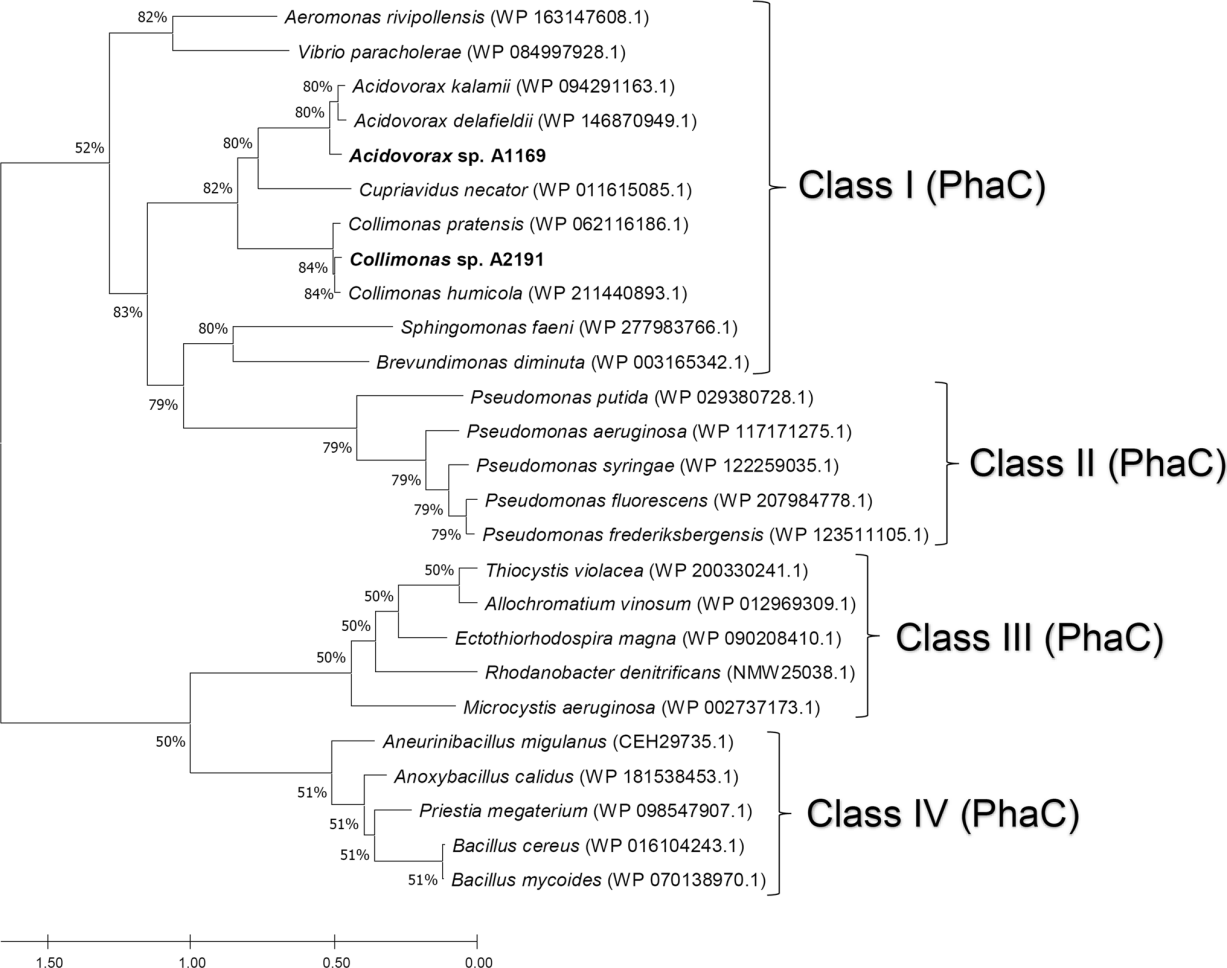
Neighbor-joining phylogenetic tree based on phaC (polyhydroxyalkanoic acid synthase) amino acid sequences showing the position of strains A1169 and A2191 phaC sequence among other phaC sequences belonging to four synthase classes. Sequences were retrieved from the NCBI database. Bar shows the substitutions per amino acid position. Numbers at nodes are bootstrap percentages based on the neighbor-joining algorithm

**Fig. 3:**
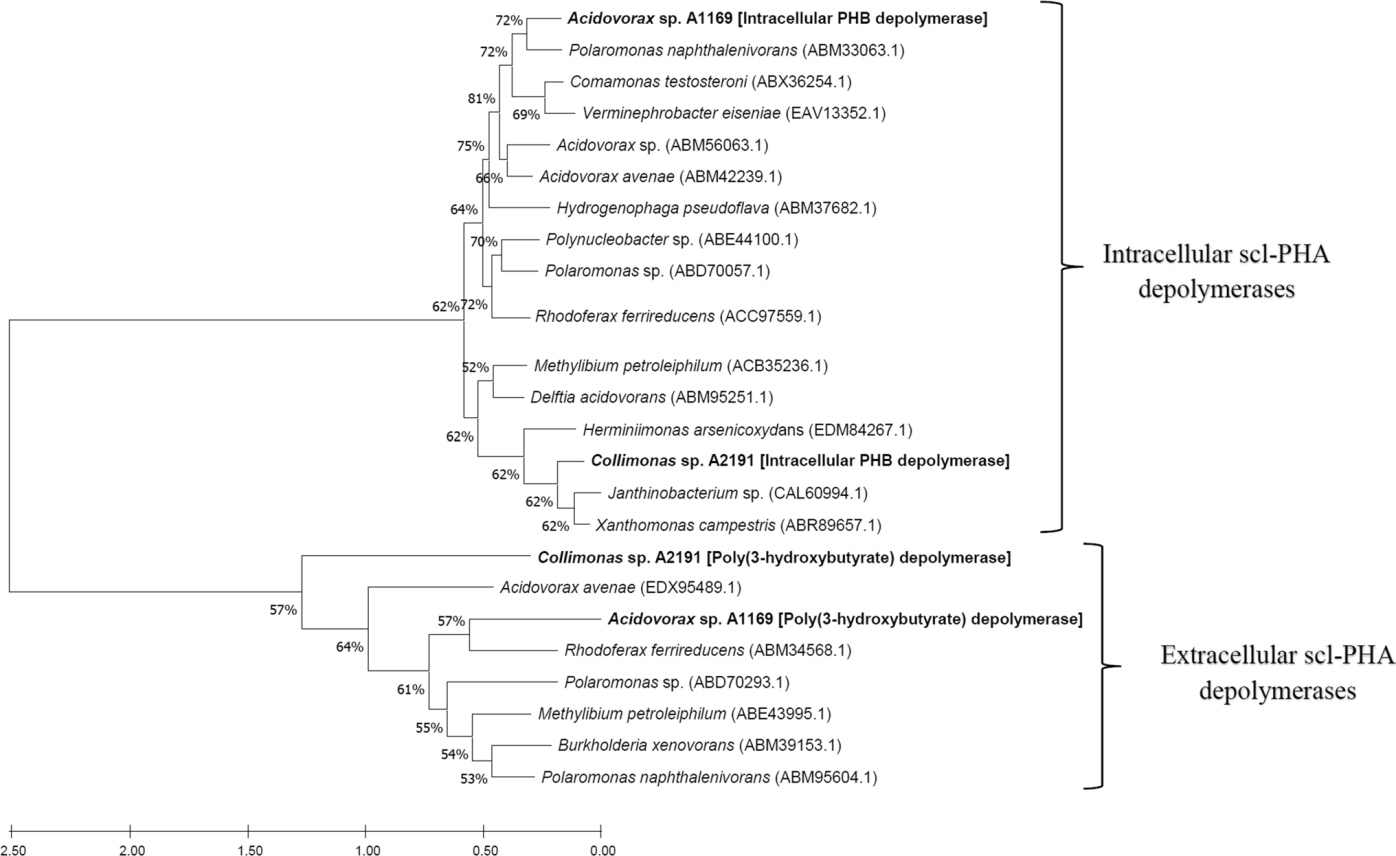
Neighbor-joining phylogenetic tree based on PHA_SCL_ depolymerases amino acid sequences showing the positions of two PHA_SCL_ depolymerases found within the genome of strains A1169 and A2191. Sequences were retrieved from the NCBI database and their identity as internal or external depolymerases was confirmed with the PHA Depolymerase Engineering Database (Knoll et al. 2009). Bar shows the substitutions per amino acid position. Numbers at nodes are bootstrap percentages based on the neighbor-joining algorithm

**Fig. 4:**
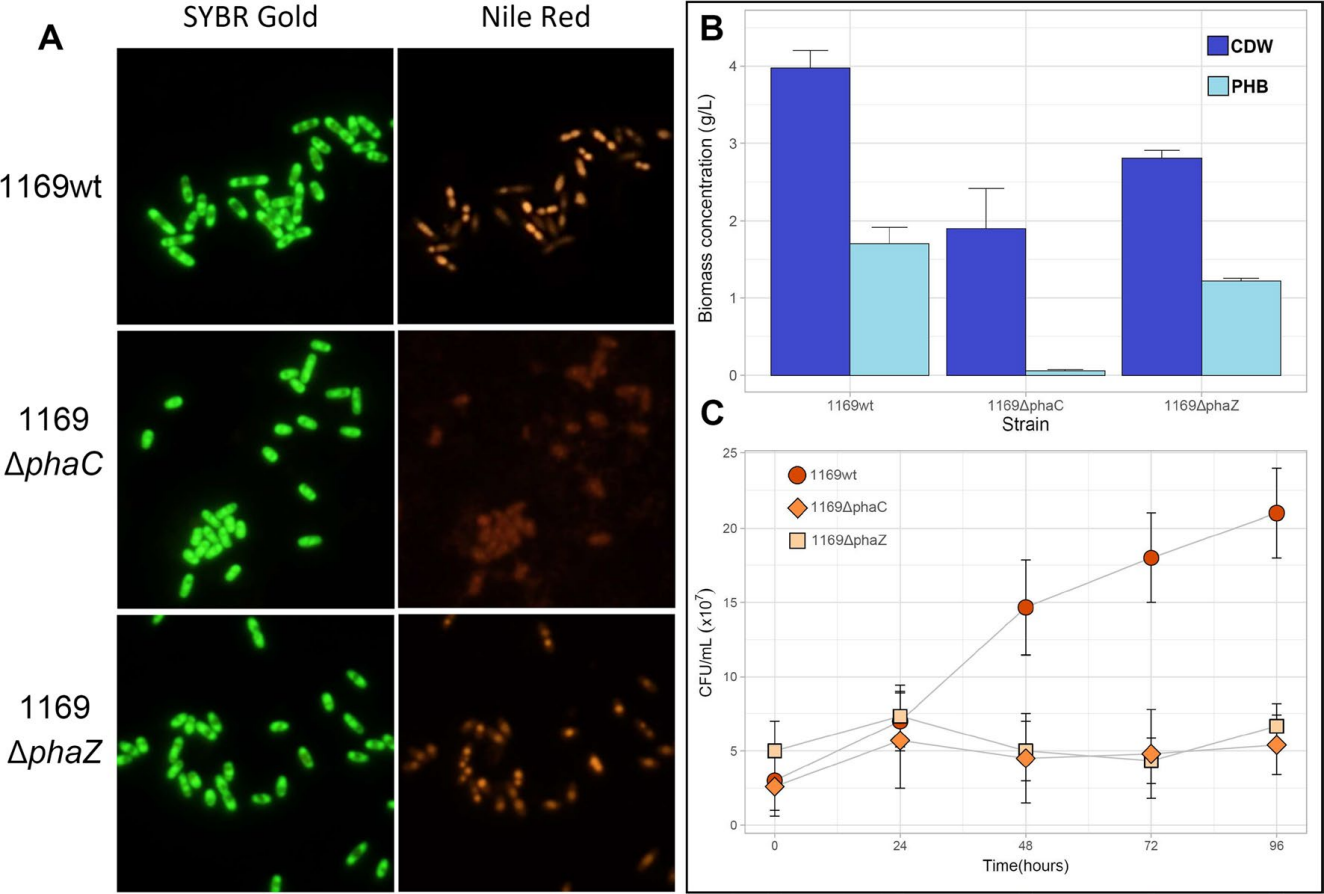
Polyhydroxybutyrate accumulation characteristics of *Acidovorax* sp. A1169 wild-type strain and its knock-out mutants. **A** Bacterial cell fluorescence (whole cell) in blue light excitation (450–490 nm) after SYBR Gold staining (left image) and in green light (PHB granule) excitation (510–560 nm) after Nile Red staining (right image). **B** Comparison of biomass concentration between strains cultivated in the same, PHB accumulation conditions. CDW-cell dry weight (dark blue), PHB-polyhydroxybutyrate (light blue). **C** Cell abundance dynamics of *Acidovorax* sp. A1169 wild-type strain and its gene knock-out mutants in carbon source-lacking mineral medium. The cells were introduced into the medium after a 96-h incubation in PHB accumulation-inducing conditions

**Fig. 5:**
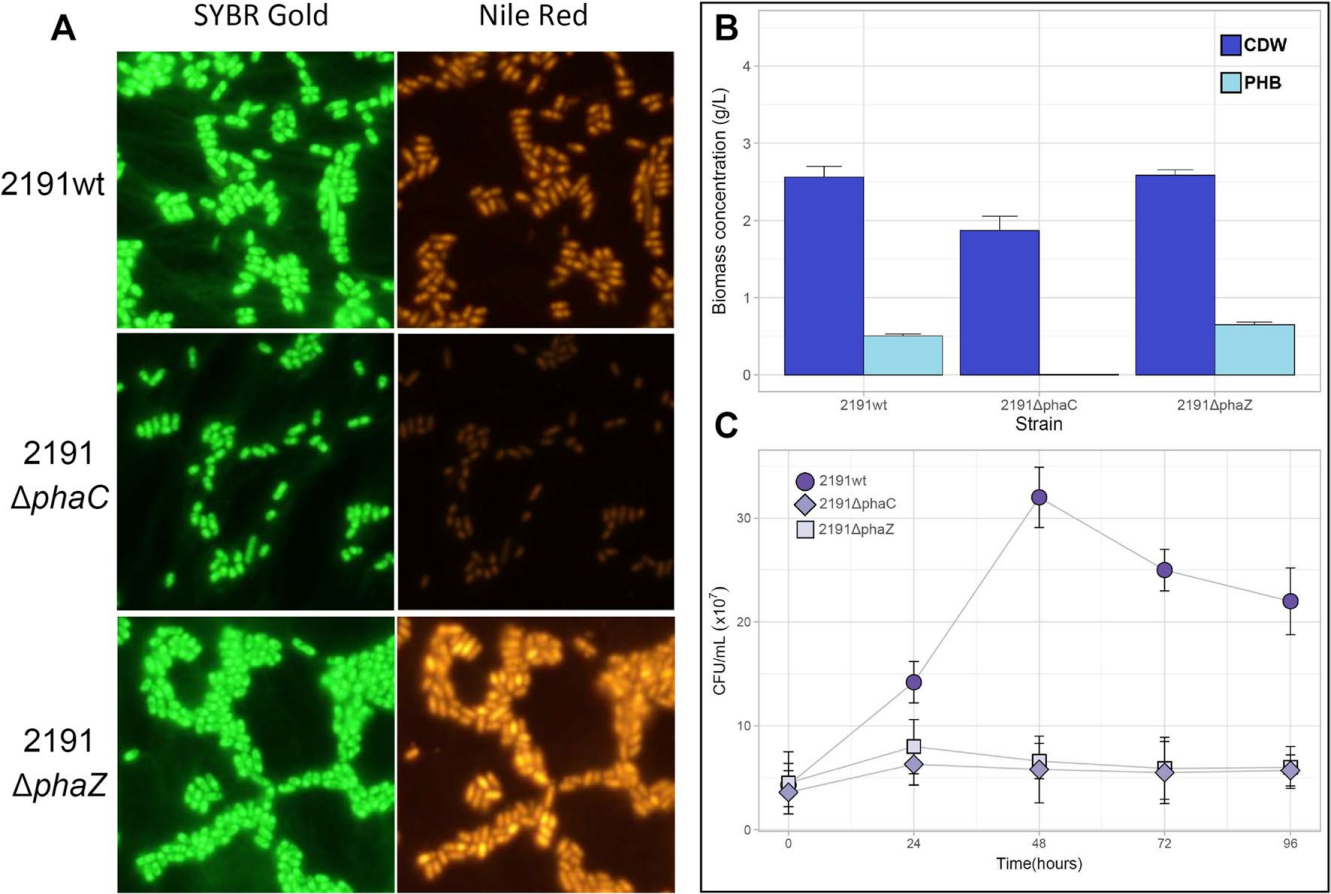
Polyhydroxybutyrate accumulation characteristics of *Collimonas* sp. A2191 wild-type strain and its knock-out mutants. **A** Bacterial cell fluorescence (whole cell) in blue light excitation (450–490 nm) after SYBR Gold staining (left image) and in green light (PHB granule) excitation (510–560 nm) after Nile Red staining (right image). **B** Comparison of biomass concentration between strains cultivated in the same, PHB accumulation conditions. CDW-cell dry weight (dark blue), PHB-polyhydroxybutyrate (light blue). **C** Cell abundance dynamics of *Collimonas* sp. A2191 wild-type strain and its gene knock-out mutants in carbon source-lacking mineral medium. The cells were introduced into the medium after a 96-h incubation in PHB accumulation-inducing conditions

The strains possessed each one gene coding for the class I polyhydroxyalkanoic acid synthase as indicated by the clustering of the neighbor-joining tree of their amino acid sequences with amino acid sequences of all four classes of the PHA synthase (Fig. 2). The presence of the class I PHA synthase typical product (homopolymer poly(3-hydroxybutyrate), PHB) was later confirmed by gas chromatography as being accumulated by both strains (Figs. 4B and 5B). Similarly, according to our results, *Acidovorax* sp. A1169 and *Collimonas* sp. A2191 both possessed each single gene coding for an intracellular scl-PHA depolymerase and one gene for an extracellular scl-PHA depolymerase, which was highlighted by clustering their deduced amino acid sequences with adequate sequences from the DED database (Fig. 3).

### Characterization of the ΔphaC and Δi-phaZ mutants

*Acidovorax* sp. A1169 wild-type strain cultivated under PHA-producing conditions accumulated PHB in the form of lipophilic inclusions (Fig. 4A). It achieved a cell dry weight (cdw) of 3.97 g/L and PHB concentration of 1.7 g/L on average (Fig. 4B) corresponding to about 42% of PHB in cdw. *Acidovorax* sp. A1169Δ*phaC* mutant did not produce any intracellular inclusions that stained with the lipophilic dye Nile Red (Fig. 4A). The concentration of PHB in the culture broth was below 0.05 g/L. *Acidovorax* sp. A1169Δ*i-phaZ* deletion mutant produced intracellular granules confirmed as PHB at a concentration of 1.22 g/L. The overall cell dry mass was however lower than that of the wild-type strain at a concentration of 2.81 g/L (Fig. 4B), while the PHB content in this deletion mutant reached about 43% of cdw. Based on the CFU count only *Acidovorax* sp. A1169 wild-type strain could access the carbon stored in its PHB granules and proliferate under carbon starvation conditions at 15 °C, while the cell concentration of both deletion mutant strains remained relatively stable after inoculation (Fig. 4C). *Collimonas* sp. A2191 and its deletion mutants behaved similarly (Fig. 5). No granules were visible in the cells of strain A2191Δ*phaC* after incubation in the PHB-production medium, while in the *Collimonas* sp. A2191 wild-type and Δ*i-phaZ* strains, the granules were clearly visible after Nile Red staining and green light exposure (Fig. 5A). The concentration of PHB in the dry cell mass of the A2191 wild-type strain was 0.5 g/L (19.7% of cdw) and in the A2191Δ*phaC* deletion mutant PHB was almost undetectable with 0.006 g/L. Contrary to the result of *Acidovorax* sp. A1169Δ*i-phaZ*, the deletion of the intracellular scl-PHA depolymerase in A2191 slightly increased its PHB yield with 0.65 g/L (25% of cdw) being produced compared to the 0.5 g/L of the wild strain (Fig. 5B). The carbon starvation proliferation assay showed that only A2191 wild-type could increase in numbers in those conditions, while the mutated strains could not (Fig. 5C).

### Stress survival of the psychrophilic strains and their mutants

**Fig. 6:**
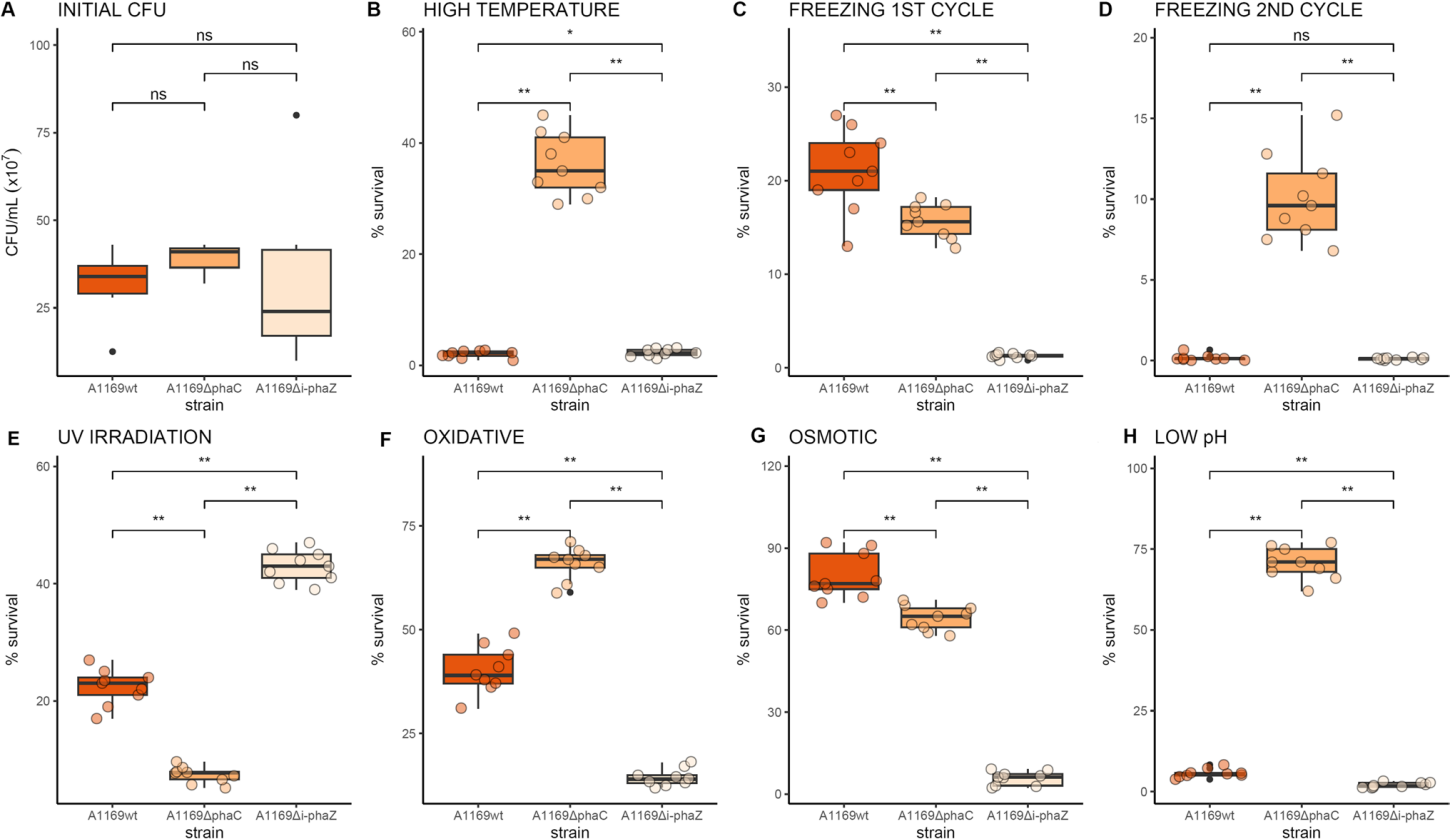
Comparison of growth (initial CFU) and survival rates (% of initial CFU) between *Acidovorax* sp. A1169 wild-type strain and its knock-out mutants. **A** CFU after a 96-h incubation in PHB accumulation-inducing conditions — initial CFU. **B** Percentage of cells surviving high-temperature exposure. **C** Percentage of cells surviving one freeze–thaw cycle. **D** Percentage of cells surviving two freeze–thaw cycles. **E** Percentage of cells surviving UV exposure. **F** Percentage of cells surviving exposure to oxidative stress. **G** Percentage of cells surviving exposure to osmotic stress. **H** Percentage of cells surviving low pH exposure. ns – not significant. *Significant at *p* < 0.05

Initial CFU numbers indicated that A1169 wild-type and its mutants achieved comparable cell concentrations in PHB production conditions, as there were no significant differences in this regard between the strains with A1169 achieving on average 3.1 × 10^8^ CFU/mL, A1169Δ*phaC* — 3.9 × 10^8^ CFU/mL, and A1169Δ*i-phaZ* — 3.3 × 10^8^ CFU/mL (Fig. 6A). High-temperature exposure caused a substantial drop in cell viability of A1169 wild-type and A1169Δ*i-phaZ* strain to 2% and 2.3% of the initial CFU, respectively. The survival of A1169Δ*phaC* strain was the highest with 36% of cells surviving the temperature shock (Fig. 6B). The first freeze–thaw cycle was best managed by the A1169 wild-type strain with 21% of cells surviving. For the A1169Δ*phaC* mutant 15.7% of the cells survived, while the cells of A1169Δ*i-phaZ* strain had only a 1.3% survival score on average (Fig. 6C). The second freeze–thaw cycle left the wild-type strain and the Δ*i-phaZ* strain with similar average viability of 0.16% and 0.11%, respectively, while the Δ*phaC* mutant displayed a cell survival of 10% of the initial CFU numbers (Fig. 6D). Exposure to UV radiation had a significantly different effect on each of the strains. A1169Δ*i-phaZ* was the most resistant with 43% of cells being viable on average post-exposure. The wild-type strain had a 22.3% survival rate while only 7.4% of the Δ*phaC* mutant cells survived the UV exposure (Fig. 6E). The oxidative agent exposure was managed best by the A1169Δ*phaC* strain with 65% of the initial cell numbers present on average, followed by A1169 wild-type (39% survival on average) and A1169Δ*i-phaZ* (14.4% survival on average) (Fig. 6F). Osmotic stress was most harmful to the A1169Δ*i-phaZ* strain leaving only 5.8% of cells in a viable and cultivable state. The wild-type and Δ*phaC* strains had a survival score of 79% and 64% of initial CFU numbers, respectively (Fig. 6G). Exposure to low pH had a detrimental effect on the wild-type strain and the Δ*i-phaZ* deletion mutant, where on average 5.7% and 2.0% of cells survived, respectively. The A1169Δ*phaC* mutant had a surprisingly high survival rate of 70.5% on average (Fig. 6H).

**Fig. 7:**
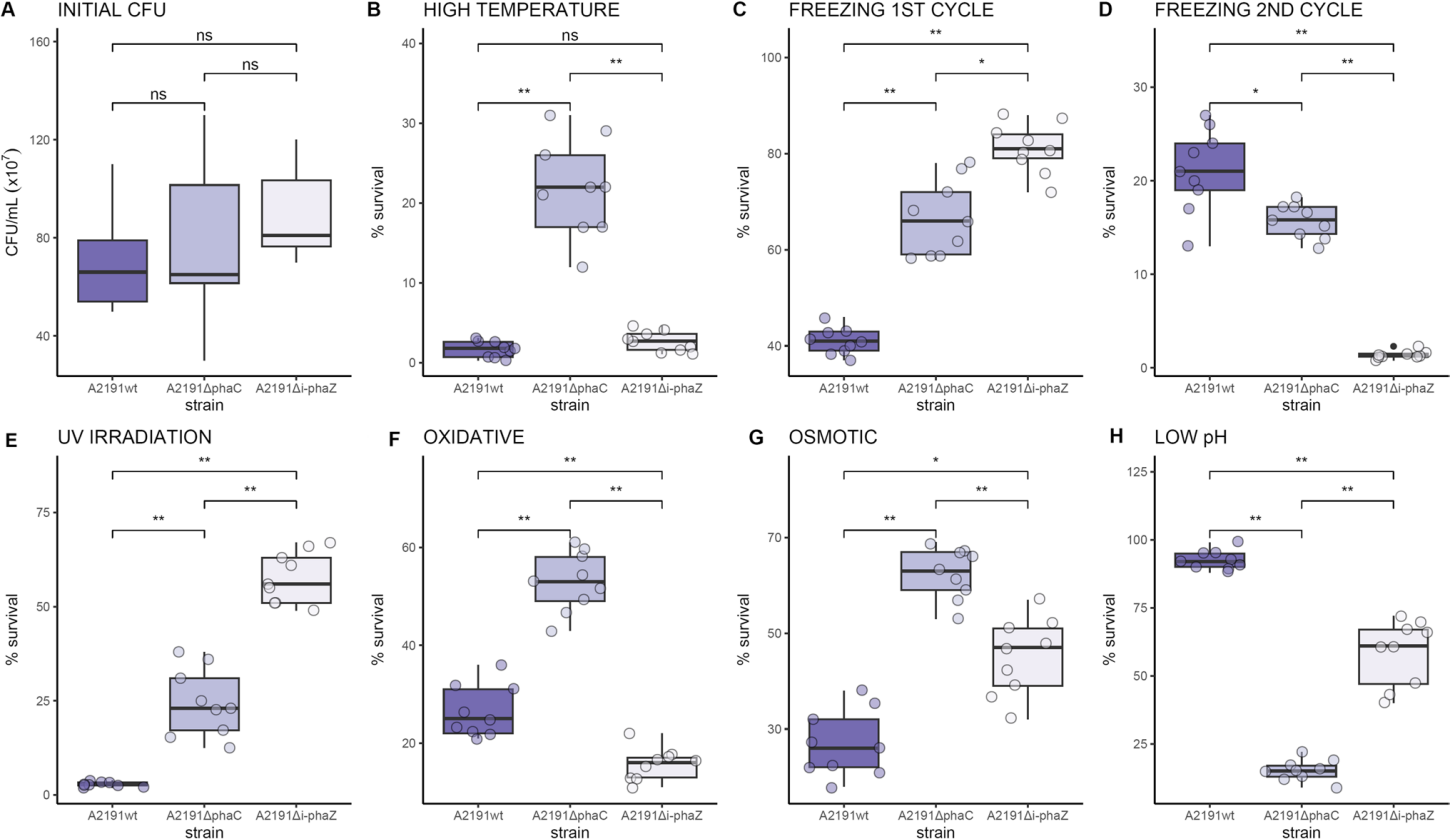
Comparison of growth (initial CFU) and survival rates (% of initial CFU) between *Collimonas* sp. A2191 wild-type strain and its knock-out mutants. **A** CFU after a 96-h incubation in PHB accumulation-inducing conditions — initial CFU. **B** Percentage of cells surviving high-temperature exposure. **C** Percentage of cells surviving one freeze–thaw cycle. **D** Percentage of cells surviving two freeze–thaw cycles. **E** Percentage of cells surviving UV exposure. **F** Percentage of cells surviving exposure to oxidative stress. **G** Percentage of cells surviving exposure to osmotic stress. **H** Percentage of cells surviving low pH exposure. ns – not significant. *Significant at *p* < 0.05

The initial cell concentrations of *Collimonas* sp. A2191 wild-type and its mutants were comparable to one another after incubation in PHB-producing conditions, achieving up to 1.2 × 10^9^ cells per mL (Fig. 7A). High-temperature exposure decreased cell viability to an average of 1.7% for the wild-type A2191 strain, 21.2% for the Δ*phaC* mutant, and 2.7% for the strain lacking the *i-phaZ* gene (Fig. 7B). Best survival of a single freeze–thaw cycle was achieved for the A2191Δ*i-phaZ* mutant, with 81.1% of cells surviving on average, followed by the Δ*phaC* mutant (66.5% cells surviving) and the wild-type strain with 41% of cells alive and cultivable (Fig. 7C). The second freeze–thaw cycle saw the situation reversed. The wild-type strain had the highest survival rate (21% on average), followed by the Δ*phaC* mutant (15.6%) and the Δ*i-phaZ* mutant with only 1.4% survival rate on average (Fig. 7D). The wild-type strain was quite sensitive to UV irradiation with only 2.8% of cells surviving the exposure to 20 kJ/m^2^. The A2191Δ*phaC* mutant did better with 24.5% of cells surviving the exposure. The best survival rate was displayed by the A2191Δ*i-phaZ* mutant with 57.7% (Fig. 7E). Oxidative stress was best managed by the A2191Δ*phaC* strain (53% of cells surviving), followed by the wild-type strain (26.4% survival rate) and the *i-phaZ* gene lacking strain (15.8%) (Fig. 7F). When challenged with sudden osmotic pressure changes the examined strains of A2191 showed significant differences in survival rates. Of the A2191Δ*phaC* strain cells, 62.4% managed to endure the exposure, while 45% and 26% of cells survived for the Δ*i-phaZ* and wild-type strains, respectively (Fig. 7F). Low pH exposure caused the initial CFU to drop to 92.4% for the wild-type A2191, 15.3% for the Δ*phaC* mutant, and 58.6% on average for the Δ*i-phaZ* mutant (Fig. 7G).

## Discussion

The protective role of PHAs has been proven on many occasions and considered one of the major factors enhancing the robustness of prokaryotic cells (Müller-Santos et al. 2021, and articles therein). Its effectiveness is dependent on an active cycle of polymerization and depolymerization where PHB granules act as a storage for the release of 3HB molecules, known to have many protective properties by themselves (Obruca et al. 2020; Ren et al. 2009). The PHB/3HB cycle also fuels other stress-alleviating cellular mechanisms by providing the necessary reductive force (NADH) needed by many processes (Ayub et al. 2009). These processes are mainly centered around removing reactive oxygen species (ROS). Enzymes involved in intracellular ROS production prevention and lipid peroxidation elimination include superoxide dismutase (SOD), catalase, and peroxidases. A crucial component of all antioxidant activity in a biological system is the H_2_O_2_ scavenging activity. NADH peroxidase eliminates potentially toxic H_2_O_2_ under aerobic growth conditions. The products of these reactions are NAD^+^ and H_2_O. The enzyme may also protect against exogenous H_2_O_2_ (Kushkevych et al. 2023).

The data obtained from the two psychrophiles in this experimental setup indicate that an active PHB–3HB exchange in the cells helps them survive the freezing-thaw cycle, which is a common feature of polar region habitats (Thomas et al. 2008). Freezing affects living cells on many levels. Ice crystal formation that is initiated outside the cell causes osmotic stress by concentrating the extracellular solutes inducing the freeze-dehydration phenomenon. Intracellular ice formation, on the other hand, may cause cell membrane disruption (Fuller 2004). The freezing process is also accompanied by the generation of reactive oxygen species (ROS) (Baek and Skinner 2012). In several studies, the cryoprotective role of PHB as well as its 3HB monomers was indicated (Obruca et al. 2016b; Slaninova et al. 2023). Strains with a functional PHB cycle generate a substantial amount of intracellular 3HB, contrary to their PHB non-accumulating mutants (Alves et al. 2020; Obruca et al. 2016a), and thus are better equipped against damaging freezing-related factors. The PHB granules themselves were speculated to alleviate some of the ice crystal formation-caused shearing stress as the native cell-bound PHB is likely to be highly flexible even at freezing temperatures (Obruca et al. 2016b). However, as *Acidovorax* sp. A1169 and *Collimonas* sp. A2191 were isolated from High Arctic’s natural environment they are likely to possess other mechanisms to cope with the frequent freeze–thaw cycle. This might explain the still considerable survival rate of the Δ*phaC* and Δ*i-phaZ* mutants. Low molecular cryoprotective solutes (amino acids, sugars, and sugar alcohols) and high molecular substances (polysaccharides, cold-shock proteins) are known to increase the survival of freeze–thaw exposed psychrophile cells (D’Amico et al. 2006). A functional PHB cycle was also advantageous in coping with osmotic fluctuation assay in this study for *Acidovorax* sp. A1169. The mechanism behind this phenomenon was proposed to involve the 3HB compound to act as a compatible solute, preventing intracellular water from being drawn out of the cell, but also the PHB polymer may act as a scaffold, supporting cell morphology and membrane continuity during plasmolysis (Obruca et al. 2017; Sedlacek et al. 2019b). PHA synthesis is a widely observed trait in halophiles, particularly in extremely halophilic Archaea (Obruča et al. 2022). This suggests that the accumulation of PHA may serve as an adaptive strategy in response to high-salinity environments. The mere presence of PHA granules leads to a reduction in intracellular space, effectively concentrating the cytoplasm. This, in turn, renders the cell less vulnerable to hyperosmotic conditions. As a result, the osmo-protective effect of PHA granules becomes more pronounced with higher intracellular PHA content. Consequently, it is reasonable to assume that this effect is significantly more pronounced in strong PHA accumulators, such as *Acidovorax* sp. A1169, compared to moderate PHA producers like *Collimonas* sp. A2191.

Many stress-challenge assays were best survived by the Δ*phaC* mutants, which is not in agreement with the observation made so far (summarized in Müller-Santos et al. 2021 and Obruca et al. 2020). However, it should be pointed out that none of such experiments to date was done with psychrophiles at low-temperature conditions. This might be the cause for those unusual findings. There is a consensus that low-temperature-growing bacteria are burdened with an increased concentration of oxidative compounds (Pischedda et al. 2018). Therefore, psychrophiles should be naturally adapted to cope with chronic oxidative stress (De Maayer et al. 2014). A severe case of PHB-synthesis apparatus involvement in protection against ROS was demonstrated by Ayub et al. (2009) where a seemingly psychrotolerant, PHB-producing *Pseudomonas* sp*.* strain, was unable to grow at 10 °C after the inactivation of its PHA synthase and consequently the disruption of the PHB/3HB cycle. It was shown that the mutant had a low NADH/NAD^+^ ratio which in turn meant low activity of NADH-dependent ROS scavenging enzymes like the metalloenzyme superoxide dismutases (SODs) (Johnson and Hug 2019). However, the Δ*phaC* mutants of *Acidovorax* sp. A1169 and *Collimonas* sp. A2191 were able to grow at low temperatures (10–15 °C) and they both showed superior survival rates after exposure to oxidative and heat-based stressors. This suggested that those mutants had higher levels of enzymes that protect against ROS than the wild-type strain. Presumably, the destruction of the PHB cycle caused the level of generated ROS to be higher than the intrinsic capability of cells to detoxify them, thus putting the cells in a state of permanent low-level oxidative stress (as there were no visible growth retardation of those cells). The levels of ROS in the cell are monitored by transcription regulators (Imlay 2015). In response to the elevated levels of ROS, DNA binding transcription regulators such as OxyR, SoxR, or OhrR activate oxidative stress-responsive genes by binding to their promoter regions (Seo et al. 2015). Such cells could therefore be better suited to cope with the acute oxidative stress applied to them in this experiment. This low-level oxidative stress might also made the cells more resistant to a heat shock. At least in *Rhodobacter sphaeroides*, *RpoH*_*II*_, the alternative sigma factor of the heat shock family, is indirectly activated by singleton oxygen (Nuss et al. 2009). It then triggers the synthesis of heat-shock proteins which prevent protein aggregation and sequestrate misfolded proteins (Ungelenk et al. 2016). Furthermore, RpoS, a general stress-response regulator, responds to oxidative stress by regulating the expression of important genes involved in multistressor resistance, which would explain the heightened resistance of the *ΔphaC* mutants to other stresses like the low pH and the osmotic challenges (Chiang and Schellhorn 2012).

In some instances, most notably after UV exposure, the Δ*i-phaZ* mutants displayed superior survival rates (for both psychrophile species). UV radiation can induce multilevel damage in single-celled organisms. Direct, and often fatal, influence on nucleic acids is based on UV absorption by DNA/RNA which causes sub-lethal mutations or leads to DNA replication arrest due to changes in its structure (Ravanat et al. 2001). Slaninova and co-workers (2018) discovered that PHA granules provide a shield-like protection of nucleoid-contained DNA molecule. By employing different spectroscopic approaches it was concluded that the granules scatter the incoming UV radiation thus diminishing its harmful effects. Presumably with the granules present and the stress-alleviating mechanisms induced by the low NADH/NAD^+^ ratio as explained before, the Δ*i-phaZ* mutant reaps the benefit of having both UV/stressor-resistance features active at the same time. However, in some cases, the Δ*i-phaZ* mutant proved the least resistant to abiotic stressors. Its vulnerability was most apparent for the 2nd freeze–thaw cycle. This could be explained by the findings of Sedlacek et al. 2019a, b, where a model was proposed, highlighting how the inactivation of the protein-based granule surface layer leads to granule coalescence, dehydration, and crystallization. The Δ*i-phaZ* mutant lacks the intracellular PHB depolymerase, which is a part of the protein assembly stabilizing the amorphous, native PHA granules. Although more research is needed, the absence of the PhaZ protein may cause the surface layer to be more denaturation-prone. Consequently, the presence of such a large, non-flexible, and biochemically inert intracellular inclusion might be a disadvantage during repeated freeze–thaw cycles, especially considering the findings of Obruca et al. (2016b).

Ultimately, PHA presence but also an active PHB/3HB cycle may be essential in survival of the most life-challenging factor present in polar regions — the frequent freeze–thaw cycle. However, the ability to synthesize and subsequently metabolize PHB probably is not the most potent mechanism in the stress-resistance arsenal of cold-loving bacteria but only one of the adaptation strategies. Although it can be intertwined with several pathways that lead to stress-response activation, alternative pathways may be engaged and a strong reaction mediated, despite crucial PHB-metabolism components missing. Yet, the stress-inducing factor intensity and diversity in High Arctic settings might be different than those applied *in vitro*, putting in doubt the long-term survival of PHB-handicapped mutants and their ecological superiority.

## Supplementary Information

Below is the link to the electronic supplementary material.

Supplementary file1 (PDF 185 KB)

## Data Availability

The data sets generated and/or analyzed in the current study are available in the NCBI repository, BioProject number PRJNA991094. Submission information can be found at https://www.ncbi.nlm.nih.gov/sra.

## Code availability

Codes used in the current study are available from the corresponding author upon reasonable request.
