# Supplementary material for: Polyhydroxyalkanoate involvement in stress-survival of two psychrophilic bacterial strains from the High Arctic
Source: Appl Microbiol Biotechnol. 2024 Mar 23;108(1):273. doi: 10.1007/s00253-024-13092-8 (PMC10960890; doi:10.1007/s00253-024-13092-8)
Supplement: Supplementary file 1 — Supplementary file1 (PDF 185 KB) [file 253_2024_13092_MOESM1_ESM.pdf]

Journal: Applied Microbiology and Biotechnology

Supplementary Material

Polyhydroxyalkanoate involvement in stress-survival of two psychrophilic bacterial strains from the High Arctic

Jakub Grzesiak[1*✉], Małgorzata Marta Rogala[1*], Jan Gawor[1], Xenie Kouřilová[2], Stanislav Obruča[2]

✉Correspondence: jgrzesiak@ibb.waw.pl

[1]Institute of Biochemistry and Biophysics, Polish Academy of Sciences, Pawińskiego 5A, 02-106 Warsaw, Poland

[2]Department of Food Chemistry and Biotechnology, Faculty of Chemistry, Brno University of Technology, Purkynova 118, 612 00 Brno, Czech Republic

*Equal contributors

Online Resource 1. DNA primers used in this study.

Primer	Primer sequence (5' – 3')	Note
A1169F<br>A1169R	TCAGGAATTCCAATGCGTCCTGCTGCTCGCGGGT (*Eco*RI)<br>TCAGGAGCTCGGCAAAGGTACCCGCCACGGAGCT (*Sac*I)	For amplifying the 472-bp upstream homologous sequence of *phaC*$_{A1169}$
C1169F<br>1196C1R	GTACGAGCTCGAAGGCAGCCACCGGGTTGGCCG (*Sac*I)<br>CCAAGCTTAGGTGCCGGTGCAGCCGAACACCAT (*Hin*dIII)	For amplifying the 348-bp downstream homologous sequence of *phaC*$_{A1169}$
A1169F<br>1196C1R	TCAGGAATTCCAATGCGTCCTGCTGCTCGCGGGT (*Eco*RI)<br>CCAAGCTTAGGTGCCGGTGCAGCCGAACACCAT (*Hin*dIII)	For the confirmation of the A1169Δ*phaC* knock-out mutant
1169Z1F<br>1169Z1R	TCAGGGATCCATAACGGAGTTTCGACCCCATGCT (*Bam*HI)<br>TCAGGAATTCGTATCGTCCGAAAAGCGCTTGAA (*Eco*RI)	For amplifying the 333-bp upstream homologous sequence of *i-phaZ*$_{A1169}$
1169Z2F<br>1169Z2R	TCAGGAATTCCGGCATCTTCAGCGGCCGGCGCTG (*Eco*RI)<br>TCAGAAGCTTGTGCGGCGGGTGCGTGGTGCCGG (*Hind*III)	For amplifying the 414-bp downstream homologous sequence of *i-phaZ*$_{A1169}$
1169Z1F<br>1169rZ	TCAGGGATCCATAACGGAGTTTCGACCCCATGCT (*Bam*HI)<br>TTCACCACCGGTTTGCTGGCGA	For the confirmation of the A1169Δ*i-phaZ* knock-out mutant
C2191F1<br>C2192R1	TCAGGGATCCATGTGCTGCAGGCGTTTGTCGATG (*Bam*HI)<br>TCAGGTCGACTCGCCATTGCATGGATTTACGTCGT (*Sal*I)	For amplifying the 542-bp upstream homologous sequence of *phaC*$_{A2191}$
C2191F2<br>C2191R2	TCAGGTCGACGTGCAGAAGGCGGCAGGACAGCTG (*Sal*I)<br>TCAGAAGCTTTACGCACAGGACCTGCCAAGGGCT (*Hin*dIII)	For amplifying the 876-bp downstream homologous sequence of *phaC*$_{A2191}$
C2191F1<br>C2191R2	TCAGGGATCCATGTGCTGCAGGCGTTTGTCGATG (*Bam*HI)<br>TCAGAAGCTTTACGCACAGGACCTGCCAAGGGCT (*Hin*dIII)	For the confirmation of the A2191Δ*phaC* knock-out mutant
Z2191F1<br>Z2191R1	TCAGGGATCCGCACACAAGTGAGCAAGGAAACAA (*Bam*HI)<br>TCAGGGTACCTTACCTCTTGGGTGGTCCAGTACA (*Kpn*I)	For amplifying the 690-bp upstream homologous sequence of *i-phaZ*$_{A2191}$
Z2191F2<br>Z2191R2	TCAGGGTACCATCTGCTGATGTCGTGGCGATCAA (*Kpn*I)<br>TCAGGAATTCGAATATTTCCCGGCGTTTTTCGGC (*Eco*RI)	For amplifying the 869-bp downstream homologous sequence of *i-phaZ*$_{A2191}$
Z2191F1<br>Z2191R2	TCAGGGATCCGCACACAAGTGAGCAAGGAAACAA (*Bam*HI)<br>TCAGGAATTCGAATATTTCCCGGCGTTTTTCGGC (*Eco*RI)	For the confirmation of the A2191Δ*i-phaZ* knock-out mutant
